# Protocol for bilateral oophorectomy in mice: a reliable and cost-effective model for promoting an obesogenic state

**DOI:** 10.1590/acb416126

**Published:** 2026-10-19

**Authors:** Juliana Andrade Pereira, Alice Crespo Ferreira, Thaísa Soares Crespo, Deborah de Farias Lelis, Vera Lúcia Lacerda Medeiros, João Marcus Oliveira Andrade

**Affiliations:** 1 Universidade Estadual de Montes Claros – Programa de Pós-Graduação em Ciências da Saúde – Montes Claros (MG) – Brazil.; 2 Universidade Estadual de Montes Claros – Faculdade de Medicina – Departamento de Fisiopatologia – Montes Claros (MG) – Brazil.; 3 Universidade Federal de Minas Gerais – Programa de Pós-Graduação em Alimentos e Saúde – Montes Claros (MG) – Brazil.

**Keywords:** Ovariectomy, Obesity, Menopause, Models, Theoretical

## Abstract

**Purpose:**

To describe a low-cost bilateral oophorectomy technique and evaluate its effects on adiposity and metabolic alterations in female mice.

**Methods:**

An experimental design was used with 16 female mice divided into two groups: control (laparotomy; n = 8) and oophorectomy (OOF; n = 8). After anesthetic induction and antisepsis, the procedure involved a medial abdominal incision, identification, and removal of the ovaries, and closure of the abdominal cavity. Animals were monitored for 12 weeks, assessing body weight, food intake, mortality, surgical complications, and biochemical parameters.

**Results:**

The animals in the OOF group exhibited significant increases in body weight and visceral adiposity, despite consuming similar amounts of food and energy compared to the control group. Moreover, the OOF group exhibited alterations in biochemical metabolic parameters, characterized by elevated levels of total cholesterol, low-density lipoprotein cholesterol (LDL-c), and glucose. No mortality was observed among the operated animals. In addition, no early or late postoperative complications were detected in either group. The average duration of the surgical procedure was approximately 7.45 minutes per mouse.

**Conclusion:**

The bilateral oophorectomy protocol is simple, low-cost, and requires minimal infrastructure. It represents a reliable experimental model for promoting an obesogenic state and metabolic alterations in female mice.

## Introduction

Obesity is considered one of the major global public health problems of the 21st century. In 2022, 2.5 billion adults aged 18 and older were overweight, including over 890 million adults who were living with obesity. This corresponds to 43% of adults aged 18 and over (43% of men and 44% of women) who were overweight^1,2^. The etiology of obesity is multifactorial, involving genetic, epigenetic, and environmental factors (such as hypercaloric diets and sedentarism)^3^. Obesity is characterized by excessive body fat deposition, resulting from an imbalance between energy intake and expenditure^4^.

In terms of genetic, biomolecular and epigenetic factors associated with obesity, the studies with animal experiments, especially with rats and mice, are of paramount importance^5-7^. There are several models of obesity induction already accepted and widely used in the world scientific literature, including surgical approaches, such as bilateral oophorectomy^8-10^.

The ovaries develop an important role in the production of sex hormones, estrogen and progesterone, which are involved in several body processes^11-13^. The bilateral oophorectomy consists in the surgical removal of the ovaries, leading to the sudden decrease in the production of progesterone and estrogen, which induces menopause^10^. This new hormonal profile predisposes, at the metabolic level, to several alterations, among them, an increase of body adiposity, especially visceral, decrease in bone mass, and alterations in the lipid and glycemic profiles^14-16^.

In the world scientific literature, several papers have adopted bilateral oophorectomy as a methodological procedure. However, only a few studies aimed to detail the surgical technique, thus hindering its standardization and replication in other study centers worldwide. Therefore, the present study aimed to describe, in a thorough and easily replicable manner, this low-cost technique, and to evaluate its effectiveness in promoting an obesogenic state and metabolic alterations in female mice. We hypothesized that this technique would effectively induce obesity in female mice and represent a reliable and accessible alternative for obesity induction in this experimental model.

## Methods

### Animals and diets

Eight-week-old female Swiss mice from the Universidade Estadual de Montes Claros (Unimontes), Montes Claros (MG), Brazil, were included in our study. The animals were randomized into two groups:

- Control group: laparotomy (n = 8);
- Oophorectomy group: OOF (n = 8).

The animals were kept in individual cages and housed under a 12-h light/dark cycle, temperature 22°C ± 2°C, with free access to food and water. All animals were placed on a standardized diet, composed by 54% of carbohydrates, 26% of proteins, 8% of lipids, and 3.2 kcal/g (Labina Presence, Brazil) for 12 weeks. The study followed all ethical principles and protocols for the use of animals in scientific research.

All stages of the study were carried out at the Health Research Institute of the Graduate Program in Health Sciences at Unimontes.

### Presurgical preparation and procedures

Presurgical preparation involved the use of sterile surgical instruments and materials specifically suited for delicate incisions and suturing techniques in small animals. The instrument set comprised scalpel handle no. 3, anatomical rat tooth forceps (10 cm), Mayo Hegar needle holder (18 cm), Mixter forceps (16 cm), Kelly curved hemostatic forceps (14 cm), straight Iris scissors (11.5 cm), curved Iris scissors (11.5 cm), and a Farabeuf retractor (0.7 cm × 10 cm). All instruments were subjected to thorough manual or ultrasonic cleaning, sterilized, and subsequently wrapped to prevent contamination and mechanical damage.

Animals were maintained under controlled conditions at 23°C on a 7 a.m.–7 p.m. light–dark cycle, with *ad libitum* access to food and water. A preoperative fasting period of six hours was observed.

General anesthesia was induced intraperitoneally using ketamine hydrochloride (100 mg/kg body weight) combined with xylazine (10 mg/kg body weight), diluted in sterile double-distilled water and administered with an insulin syringe. The mean induction time ranged from 3 to 5 minutes. If necessary, a supplementary dose corresponding to one-third of the initial ketamine dose was administered. Throughout the procedure, animals’ vital parameters were monitored, including respiratory rate, nociceptive reflexes, and spontaneous movements. Following induction, animals were positioned under a heating source to assist in maintaining normothermia.

Pre-emptive analgesia was administered with ketoprofen (50 mg/mL; 5 mg/kg, intramuscularly) 10 minutes prior to incision, followed by a second dose at 12 hours. An additional dose of ketoprofen was scheduled if the animal exhibited signs of pain at 24 hours. Furthermore, acetaminophen/paracetamol (200 mg/mL; 2 mg/mL in drinking water) was provided *ad libitum*. Indicators of pain included reduced food and water intake associated with weight loss, piloerection, decreased interaction with the environment, alterations in vocalization (spontaneous or stimulus-evoked), oculonasal discharge, changes in respiratory pattern, tremors, seizures, prostration, and self-mutilation behaviors. Recovery and postoperative monitoring were continuous for 48 hours.

After anesthetic induction, animals were positioned supine on a sterile cotton surgical field placed on a 70-cm-high bench and secured at the limbs with adhesive tape (Fig. 1a). Antisepsis of the abdominal region was then performed using 1% alcoholic chlorhexidine, followed by scrubbing with sterile gauze soaked in degerming solution.

**Figure 1:**
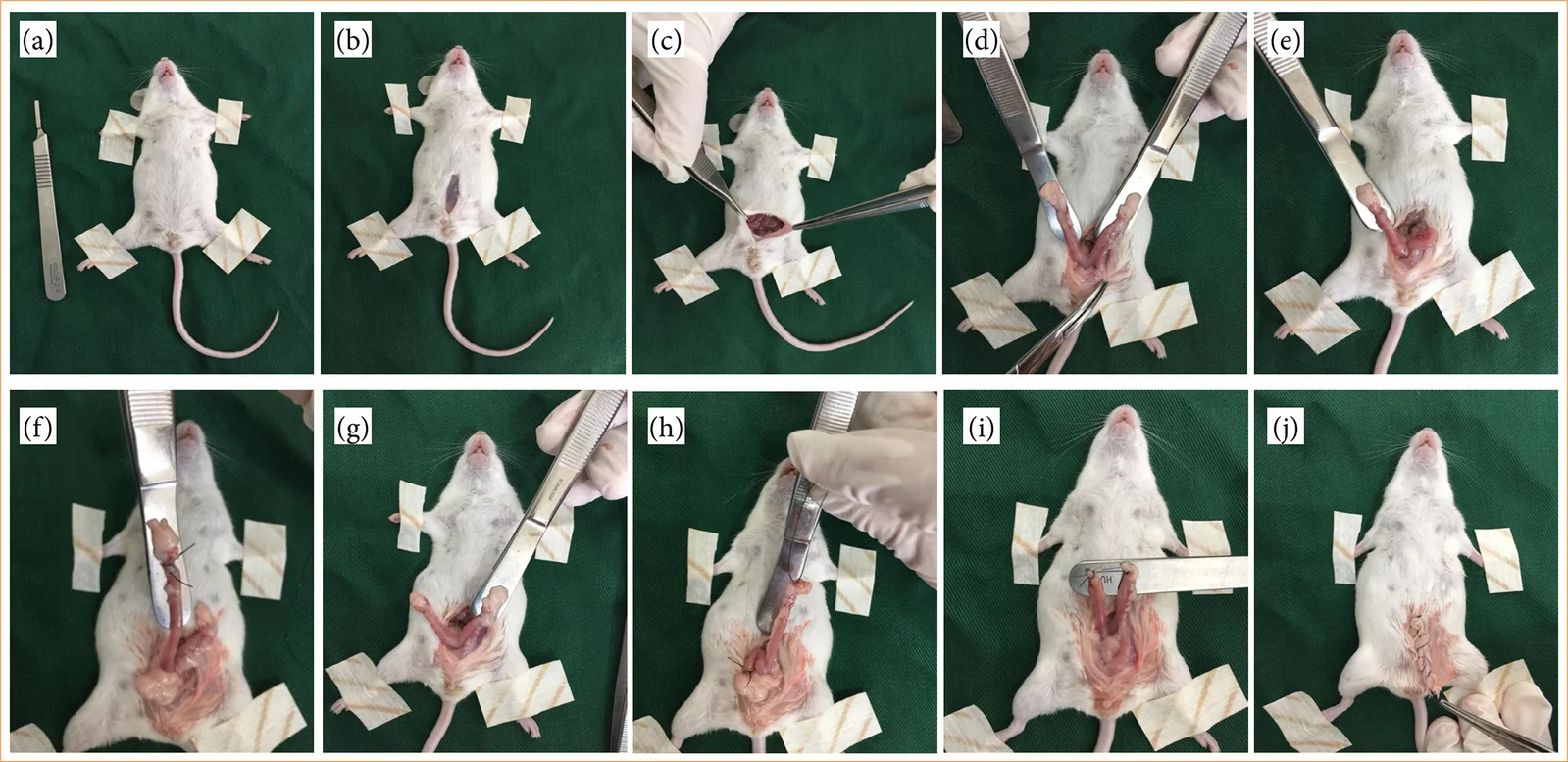
Sequential steps of bilateral oophorectomy in mice. (a) Positioning of the anesthetized animal in dorsal decubitus on a sterile surgical field with limb fixation. (b) Performance of a median laparotomy. (c) Opening of the peritoneal cavity after peritoneal incision. (d) Identification and exteriorization of the ovaries and uterus through the abdominal incision. (e and f) Ligation of the uterine horn, excision of the ovary and periovarian adipose tissue, and verification of hemostasis on the right side. (g and h) Repetition of the same surgical procedure on the contralateral side. (i) Inspection of the abdominal cavity and confirmation of hemostasis after bilateral ovary removal. (j) Closure of the abdominal wall with continuous single-layer suturing.

Source: Elaborated by the authors.

The surgical procedure was initiated with a median incision (median laparotomy) of approximately 1 cm, made by a no.11 scalpel blade (Fig. 1b). The peritoneum was then incised to the same length, exposing the abdominal cavity (Fig. 1c). Gentle manipulation of the intestinal loops with anatomical forceps allowed identification and exteriorization of the ovaries and uterus through the same incision (Fig. 1d).

The ligation site of the right uterine horn was identified using a hemostatic forceps, followed by a ligation with 3-0 braided silk suture and excision of the ovary along with the periovarian adipose tissue. Hemostasis was subsequently verified (Figs. 1e and 1f). The same procedure was performed on the contralateral side (Figs. 1g and 1h).

Upon completion of bilateral ovary removal, the abdominal cavity was carefully inspected, and hemostasis reconfirmed (Fig. 1i). Closure of the abdominal wall (laparorrhaphy) was performed with a continuous single-layer suture using 4-0 monofilament nylon (Fig. 1j). The surgical wound was irrigated with sterile 0.9% NaCl solution and gently dried with sterile gauze.

### Postoperative procedures

Postoperatively, animals were housed in individual cages with bedding composed of wood shavings and sterile cotton pads. They were kept under a focused heating lamp for six hours to stabilize body temperature, thereby preventing hypothermia and reducing the metabolic response associated with surgery. Food and water were provided *ad libitum* and made available immediately after anesthetic recovery. The postoperative analgesia protocol followed the procedures described in the “Presurgical preparation and procedures” section.

### Complications and mortality follow-up

The animals were daily accompanied during the first two weeks after the postoperative procedures, to evaluate eventual systemic, and/or operatory-wound complications. The observation was performed by a single evaluator, twice a day, observing sings of wound infection, and the animal’s general characteristics, including body weight gain/loss, food intake pattern, coat appearance, and movement during the waking period. Furthermore, during the whole experimental period, the animal’s deaths were recorded.

### Measurement of body weight and food consumption

The body weight and food consumption of the animals were measured once a week, on fixed and previously defined days and time. For body weight, the values per animal, in grams, were recorded throughout the experiment, as well as the means per group on each day of measurement. Food intake was calculated as the difference between the initial and final food weight, divided by the number of experimental days and normalized by the mean body weight of the group during the evaluation period.

### Euthanasia, tissue collection, and adiposity measurement

At the end of the experimental protocol, animals were deeply anesthetized using ketamine hydrochloride 100 mg/kg and xylazine 30 mg/kg intraperitoneally, and adequate depth of anesthesia was confirmed by the absence of the pedal withdrawal reflex prior to euthanasia. Animals were subsequently euthanized by guillotine decapitation in accordance with the approved institutional animal care and use protocol. Immediately after sacrifice, blood samples and white adipose tissue (gonadal, mesenteric, and retroperitoneal) samples were collected. The visceral adiposity was calculated by Eq. 1:


(1) Visceraladiposity=∑weightofgonadal,mesenteric,andretroperitonealtissues(g)/animalweight(g)]×100

### Intraperitoneal glucose tolerance test and insulin sensitivity test

The intraperitoneal glucose tolerance test (ipGTT) was performed after a 12-hour overnight fast, using an intraperitoneal injection of hypertonic glucose solution at 50% (w/v) (Samtec^®^, Brazil) at a dose of 2 g/kg body weight. Peripheral blood samples were collected via a small incision at the distal portion of the tail, with an estimated volume of 5–10 μL per collection, at time points 0 (baseline), 15, 30, 60, and 120 minutes after injection for glucose measurement using an Accu-Chek glucometer (Roche Diagnostics, United States of America).

The intraperitoneal insulin sensitivity test was conducted in the morning under fed conditions. Animals received insulin (100 IU/mL; Insulin N^®^, Novo Nordisk) at a dose of 0.75 IU/kg body weight, and blood glucose levels were measured at time points 0 (baseline), 15, 30, and 60 minutes after injection. Blood collection and glucose measurement procedures were performed as described for the ipGTT.

### Blood biochemical analysis

The serum was separated from the fresh blood samples by centrifugation. Total cholesterol, high-density lipoprotein cholesterol (HDL-c), triglyceride, insulin, and glucose levels were evaluated using specific kits (In Vitro Diagnostica, Brazil). Low-density lipoprotein cholesterol (LDL-c) levels were calculated using the formula proposed by Friedewald et al.^17^.

### Histological analysis

Ovarian samples collected at euthanasia were immediately fixed in 10% buffered formalin, followed by dehydration in graded ethanol solutions, clearing in xylene, and paraffin embedding for histological processing. The tissue’s sections (5 μm) were stained with hematoxylin and eosin (H&E) using standard techniques. The samples were examined using an optical microscope Evolution LC color light camera (Media Cybernetics, United States of America), which captured images.

### Statistical analysis

Data were expressed as means ± standard error of the mean (SEM) and analyzed using GraphPad Prism version 5.0 (GraphPad Software, United States of America). Data normality was assessed using the Kolmogorov–Smirnov test. Comparisons between two groups were performed using the unpaired Student’s t-test or the Mann–Whitney’s test, according to data distribution, followed by Tukey’s post hoc test. For ipGTT and intraperitoneal insulin tolerance test analyses, two-way analysis of variance (ANOVA) was applied. Differences were considered statistically significant at *p* < 0.05.

## Results

It was observed that the animals submitted to oophorectomy displayed an increase in mean body weight compared with the control group (control = 37.38 ± 1.90 *versus* OOF = 42.46 ± 3.22; *p* = 0.016) (Figs. 2a and 2b). Two-way ANOVA revealed significant main effects of group (F(1,144) = 9.855, *p* = 0.0021) and time (F(11,144) = 8.952, *p* < 0.0001), whereas the group × time interaction was not significant (F(11,144) = 0.4685, *p* = 0.920). Nevertheless, analysis of the area under the curve for body weight demonstrated a significantly greater overall body weight exposure in the OOF group compared with the control group (median area under the curve [AUC]: control = 413.5 ± 7.91 versus OOF = 452.4 ± 12.98, *p* = 0.0043) (Fig. 2b). The same tendency was maintained when body weight variation was calculated as the difference between final and initial body weight, with a greater relative percentage variation observed in the OOF group compared with the control group (control = 22.29 ± 5.87 versus OOF = 32.82 ± 9.90; *p* = 0.049) (Fig. 2c). Despite similar food consumption expressed as kcal/g body weight/day between groups (control = 0.33 ± 0.12 versus OOF = 0.35 ± 0.13; *p* = 0.471), the OOF group presented greater visceral fat accumulation compared with the control group (control = 9.63 ± 0.99 versus OOF = 11.70 ± 1.50; *p* = 0.034) (Figs. 2d and 2e). These findings indicated that oophorectomy is associated with increased body weight and visceral adiposity, without significant differences in food intake between groups.

**Figure 2:**
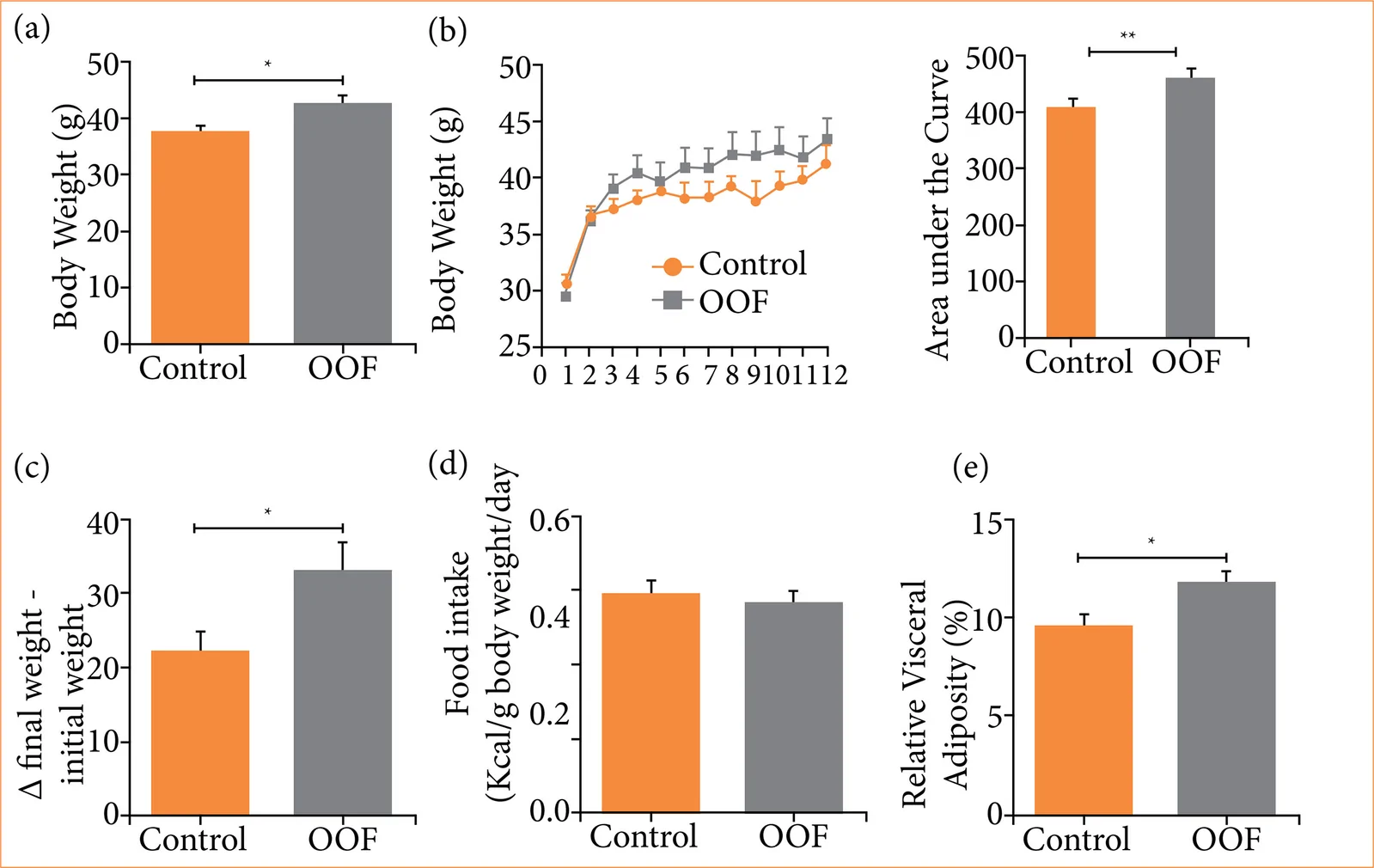
Effects of bilateral oophorectomy on body weight gain, food intake, and adiposity in mice. (a) Final body weight, (b) weekly body weight progression and respective area under the curve (AUC), (c) percentage of body weight gain relative to initial body weight, (d) food intake normalized by body weight, and (e) relative visceral adiposity are presented. Data are expressed as mean ± standard error of the mean (SEM) (n = 8 animals per group). Data were analyzed using Student’s t-test or Mann-Whitney’s test, except for weekly body weight progression, which was analyzed using two-way analysis of variance (ANOVA) followed by Tukey’s post hoc test.

*p < 0.05: statistically significant differences between groups; **p < 0.01: statistically significant differences between groups. Source: Elaborated by the authors.

Regarding the metabolic biochemical parameters, higher levels of total cholesterol (control = 80.33 ± 2.37 *versus* OOF = 147.70 ± 5.53; *p* < 0.0001) and LDL-c (control = 45.25 ± 3.50 *versus* OOF = 100.01 ± 5.65; *p* < 0.0001) were observed (Figs. 3a and 3b). No statistically significant differences were found for HDL-c and triglycerides (Figs. 3c and 3d). Concerning the glycemic profile, insulin sensitivity and glucose tolerance tests did not differ between groups (Figs. 3e and 3f). The AUC did not differ significantly between groups in either the intraperitoneal insulin sensitivity test (control = 4,908.8 ± 226.8 *versus* OOF = 5,167.5 ± 353.3; *p* = 0.155) or the ipGTT (control = 20,856.0 ± 752.9 *versus* OOF = 21,417.0 ± 712.8; *p* = 0.396). However, insulin levels did not differ between groups, whereas glucose levels were higher in the OOF group (Figs. 3g and 3h).

**Figure 3:**
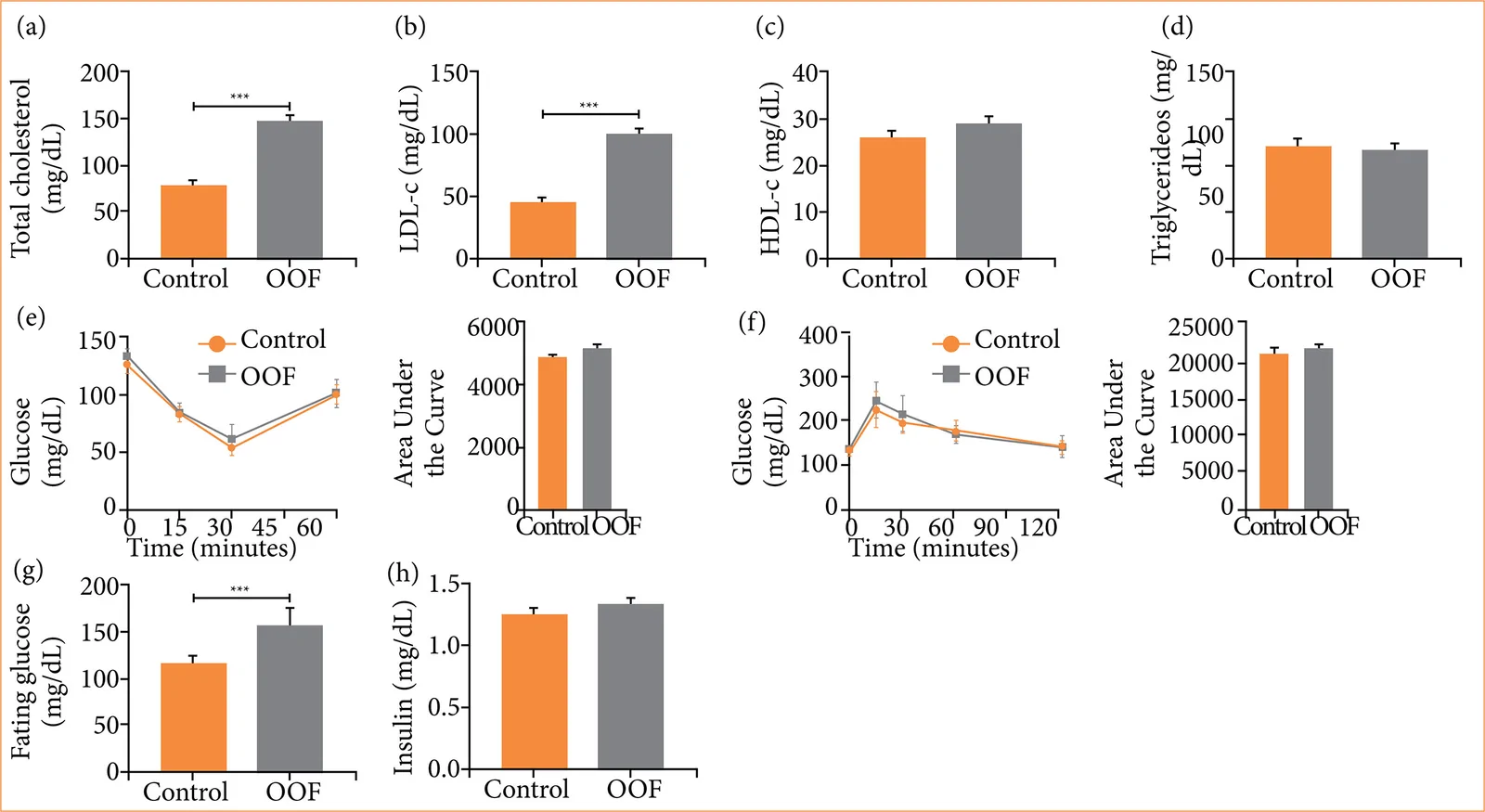
Metabolic assessment of mice subjected to bilateral oophorectomy. (a) Serum levels of total cholesterol, (b) low-density lipoprotein cholesterol (LDL-c), (c) high-density lipoprotein cholesterol (HDL-c), and (d) triglycerides are shown. (e) Blood glucose levels during the intraperitoneal insulin sensitivity test (IpIST) and the respective area under the curve (AUC). (f) Blood glucose levels during the intraperitoneal glucose tolerance test (IpGTT) and the respective AUC. (g) Fasting blood glucose. (h) Serum insulin levels. Data are expressed as mean ± standard error of the mean (SEM) (n = 8 animals per group). Data were analyzed using Student’s t-test or Mann-Whitney’s test, except for IpGTT and IpIST data, which were analyzed using two-way analysis of variance (ANOVA) followed by Tukey’s post hoc test.

***p < 0.001: statistically significant differences between groups. Source: Elaborated by the authors.

To confirm the adequacy of the surgical procedure, histological analysis of the excised tissue was performed in the experimental group. As shown in Fig. 4a, the sections demonstrated typical ovarian morphology, including preserved ovarian architecture and follicles at different developmental stages, confirming that the resected specimens corresponded to ovarian tissue and validating successful oophorectomy.

**Figure 4:**
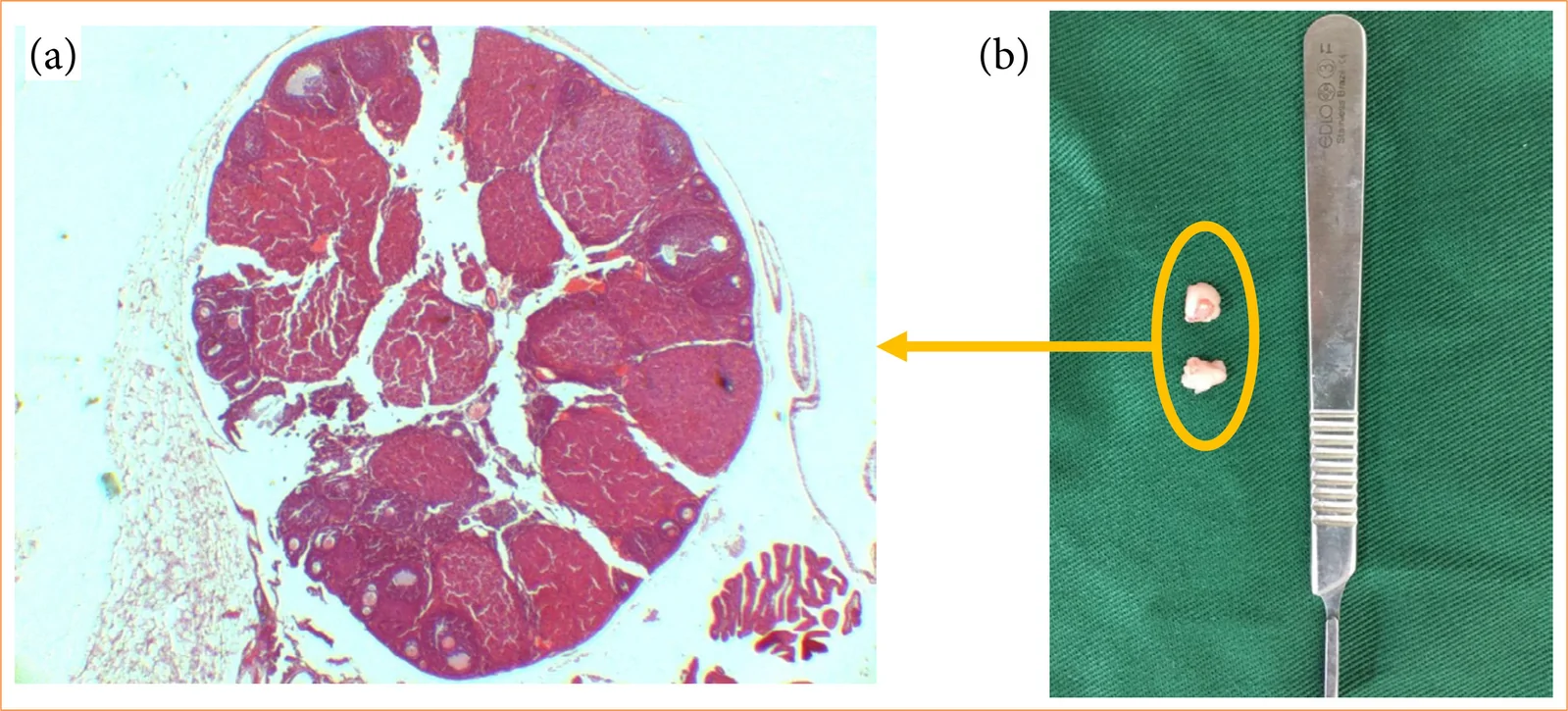
Microscopy and macroscopic appearance of a bilateral oophorectomy specimen. (a) Representative hematoxylin and eosin staining of the ovary (scale bar = 200 μm), demonstrating preserved ovarian architecture with follicles at different stages of development. (b) The freshly resected surgical specimen, containing two ovarian fragments (circled).

Source: Elaborated by the authors.

Regarding the postoperative complications follow-up, no signs of wound infection were observed, revealing a great operative wound healing process and absence of inflammatory process due to the suture thread permanence. No early or late deaths associated with the surgical procedures were observed in either group. Furthermore, the surgical procedure mean time was monitored, and lasted 7.45 minutes in the OOF group, period comprised since the median incision until the operatory wound closure.

## Discussion

The experimental model of bilateral oophorectomy in mice is widely used to investigate the physiological consequences of estrogen deprivation, as it consistently mimics the menopausal condition. The removal of the ovaries results in an abrupt decline in circulating estrogen levels, leading to endocrine and metabolic alterations that include increased visceral adiposity, redistribution of body fat, and insulin resistance. Such changes favor the development of obesity and a metabolic phenotype like that observed in postmenopausal women.

The present study aimed to describe a reproducible and cost-effective oophorectomy model in mice. Furthermore, as previously demonstrated in the scientific literature, our findings confirmed that this model can induce characteristic body composition and biochemical changes consistent with an obesity model. In addition, this experimental design enhances the translational validity of the model, allowing for the investigation of both pathophysiological mechanisms and therapeutic strategies aimed at preventing and treating metabolic complications associated with menopause.

In our model, the animals in the OOF group exhibited significant increases in body weight and visceral adiposity, despite consuming similar amounts of food and energy compared to the control group, thereby demonstrating the effectiveness of the surgical procedure in inducing obesity. Moreover, alterations in biochemical metabolic parameters were observed, including elevated total cholesterol levels and LDL-c concentrations in the OOF group. No mortality was observed among the operated animals. In addition, no early or late postoperative complications were detected in either group. The average duration of the surgical procedure was approximately 7.45 minutes per mouse.

## Conclusion

In summary, the bilateral oophorectomy proved to be easy to perform, requiring minimal infrastructure and low-cost materials. The animals exhibited excellent adaptation to the surgical procedure, with no complications or surgery-related deaths. Moreover, the oophorectomy was successful in promoting increased body weight and fat accumulation, thereby promoting an obesogenic state in the studied animals, as well as causing metabolic alterations.

## Data Availability

The data will be available upon request.
